# A randomized controlled trial to assess the efficacy and cost-effectiveness of urinary catheters with silver alloy coating in spinal cord injured patients: trial protocol

**DOI:** 10.1186/1471-2490-13-38

**Published:** 2013-07-30

**Authors:** Xavier Bonfill, David Rigau, María Luisa Jáuregui-Abrisqueta, Juana María Barrera Chacón, Sebastián Salvador de la Barrera, Carolina María Alemán-Sánchez, Manuel Bea-Muñoz, Susana Moraleda Pérez, Albert Borau Duran, Juan Ramón Espinosa Quirós, Luís Ledesma Romano, Manuel Esteban Fuertes, Ignacio Araya, Ma José Martínez-Zapata

**Affiliations:** 1 Service of Clinical Epidemiology, Hospital de la Santa Creu i Sant Pau, Calle Sant Antoni M. Claret, 167, PO: 08025, Barcelona, Spain; 2 Iberoamerican Cochrane Centre, Calle Sant Antoni M. Claret, 167, PO: 08025, Barcelona, Spain; 3 Institute of Biomedical Research (IIB Sant Pau), Barcelona, Spain; 4 CIBERESP (CIBER de Epidemiología y Salud Pública), Barcelona, Spain; 5 Department of Paediatrics, Obstetrics and Gynaecology and Preventive Medicine, Universitat Autònoma de Barcelona, Bellaterra, Spain; 6 Hospital Universitario Cruces, Plaza de Cruces s/n, PO: 48903, Barakaldo Bizkaia, Spain; 7 Hospital Universitario Virgen del Rocío, Avenida Manuel Siurot s/n, PO: 41013, Sevilla, Spain; 8 Complexo Hospitalario Universitrio A Coruña, Avenida As Xubias s/n, PO: 15006, A Coruña, Spain; 9 Complejo Hospitalario Universitario Insular Materno Infantil, Avenida Marítima del Sur, s/n. PO: 35016, Las Palmas de Gran Canaria, Spain; 10 Hospital Universitario Central de Astúrias, Calle Celestino Villamil, s/n. PO: 33006, Oviedo, Spain; 11 Hospital Universitario La Paz, Paseo de la Castellana, 261, PO: 28046, Madrid, Spain; 12 Hospital de Neurorrehabilitación - Instituto Guttmann, Camí de Can Ruti, s/n, PO: 08916, Badalona, Spain; 13 Hospital Universitario Puerta del Mar, Avenida Ana de Viya, 21, PO: 11500, Cádiz, Spain; 14 Hospital Universitario Miguel Servet, Calle Isabel la Católica, 1-3, PO: 50009, Zaragoza, Spain; 15 Hospital Nacional de Parapléjicos de Toledo, Finca de La Peraleda, s/n, PO: 45071, Toledo, Spain; 16 Departamento de Cirugía y Traumatología Maxilofacial, Facultad de Odontología - Universidad de Chile, Calle Sergio Livingstone Pohlhammer 943, Independencia, PO: 8380–492, Santiago de Chile, Chile

**Keywords:** Spinal cord injuries, Urinary tract infection, Urinary catheters, Protocol, Randomized clinical trial

## Abstract

**Background:**

Patients with non-acute spinal cord injury that carry indwelling urinary catheters have an increased risk of urinary tract infection (UTIs). Antiseptic Silver Alloy-Coated Silicone Urinary Catheters seems to be a promising intervention to reduce UTIs; however, actual evidence cannot be extrapolated to spinal cord injured patients. The aim of this trial is to make a comparison between the use of antiseptic silver alloy-coated silicone urinary catheters and the use of standard urinary catheters in spinal cord injured patients to prevent UTIs.

**Methods/Design:**

The study will consist in an open, randomized, multicentre, and parallel clinical trial with blinded assessment. The study will include 742 spinal cord injured patients who require at least seven days of urethral catheterization as a method of bladder voiding. Participants will be online centrally randomized and allocated to one of the two study arms (silver alloy-coated or standard catheters). Catheters will be used for a maximum period of 30 days or removed earlier if the clinician considers it necessary. The main outcome will be the incidence of UTIs by the time of catheter removal or at day 30 after catheterization, the event that occurs first. Intention-to-treat analysis will be performed, as well as a primary analysis of all patients.

**Discussion:**

The aim of this study is to assess whether silver alloy-coated silicone urinary catheters improve ITUs in spinal cord injured patients. ESCALE is intended to be the first study to evaluate the efficacy of the silver alloy-coated catheters in spinal cord injured patients.

**Trial registration:**

NCT01803919

## Background

Most patients with spinal cord injuries (SCI) suffer from some type of neurogenic lower urinary tract dysfunction which requires the use of different strategies for voiding, including urinary catheters. Almost all patients with indwelling urinary catheters develop bacteriuria and in consequence urinary tract infections (UTI) occur frequently. Frequent UTI are bothersome for the patients, implies frequent antibiotic use and increase of resistant strains, may cause septicemia and are therefore related to a decreased health-related quality of life [1-3].

Several trials have evaluated the effectiveness of different types of indwelling urinary catheters to prevent urinary infections in hospitalized adults requiring short-term catheterization. A systematic review of randomized controlled trials revealed that silver-alloy but not silver-oxide coated catheters, were associated with a significant reduction in bacteriuria in comparison to standard catheters [4]. Another recent systematic review aimed to determine which type of indwelling urinary catheter is best to use for long-term bladder drainage in adults found that all trials were small and with methodological weaknesses. The evidence from this systematic review was not sufficient as a reliable basis for practical conclusions [5].

A large randomized clinical trial assessing three different catheters (silver-alloy-coated, nitrofural-impregnated and a PTFE-coated catheters) in hospitalized adults with acute pathologies requiring short urinary catheterization has been recently published. No clinically relevant difference was noted between the three groups [6].

Few randomized clinical trial have evaluated the effectiveness of different catheters in SCI population, most of them assessing the use of intermittent catheterization, but none using antiseptic-impregnated catheters [7-11].

We propose a clinical trial in SCI patients needing an indwelling urethral catheter to assess the effectiveness of urinary catheters with antiseptic, silver-alloy coating. The study hypothesis is that the use of these urinary catheters reduces the risk of UTI compared to the use of conventional urinary catheters.

## Methods/Design

### Study design

This study is an open, randomized, multicentre, and parallel clinical trial. Local ethics approvals have been obtained from all centres participating in this study

### Participants

Seven-hundred and forty-two participants in this study are being recruited from rehabilitation or spinal units at specialized hospitals and outpatient centres across Spain (Table 1 and ESCALE group in Additional file 1). The inclusion period is from November 2012 to December 2014.

**Table 1:** Participating centers

**Coordinating centre:**	
**Iberoamerican Cochrane Centre. Institute of Biomedical Research (IIB Sant Pau), Barcelona**	
Recruiters centers:	
1	Complexo Hospitalario Universitario A Coruña
2	Hospital Universitario Central de Asturias
3	Complejo Hospitalario Universitario Insular – Materno Infantil de Canarias
4	Hospital Universitario Cruces, Barakaldo.
5	Hospital Universitario Miguel Servet, Zaragoza
6	Hospital Universitario La Paz, Madrid
7	Hospital Nacional de Parapléjicos de Toledo
8	Hospital Universitario Virgen del Rocío, Sevilla
9	Hospital de Neurorrehabilitación - Instituto Guttmann, Badalona
10	Hospital Universitario Puerta del Mar, Cadiz

This yields the inclusion of patients with first-ever urethral catheterization and also patients with indwelling urethral catheterization as a method of voiding.

The criteria for inclusion in this study are:

1. Male or female patients with traumatic or medical spinal cord injury.

2. Age of 18 years or above.

3. Patients who need an indwelling urinary catheter as a method of bladder drainage for at least 7 days.

4. Patients who are willing to participate in the study and give their written informed consent (If a patient is unable to give written consent because of physical or mental disability, an affirmation of consent will be taken in his presence from his relative or legal guardian).

The following participants will not be included in the study:

1. Patients who can benefit from other method of bladder drainage such as intermittent catheterization, suprapubic drainage or reflex voiding; as well as those using an external collector.

2. Patients with urinary tract infection at the moment of inclusion.

3. Current antibiotic use or use within 7 days prior to inclusion.

4. Outpatients with sporadic medical examinations (less than one per month).

5. Known allergy to latex, silver salts or hydrogels.

6. Patients with surgical interventions in the urinary tract that may interfere, at the investigator criteria, with the study results.

7. Pregnant or breastfeeding woman.

Subjects eligible for the study are contacted through medical staff and are provided with information about the study (verbal and written information). Consent is then obtained for participation in the study.

### Procedures

#### Interventions

Participants are randomly allocated to intervention or control arms.

The intervention arm receives urinary catheters with antiseptic silver alloy coating (BIP Foley catheter – Silicone, Bactiguard® Infection Protection). Bactiguard® Infection Protection coating consists of noble metals such as gold, palladium and silver and its mechanism of action is to avoid the adhesion of bacteria to the inner catheter surface. All are made of full silicone.

The control arm receive standard urinary catheters commonly used in each hospital, most of them made of silicone or silicone-latex.

Trained health staff performs urethral catheterization procedure and select the most adequate catheter size. To ensure aseptic conditions they are asked to strictly follow the current protocol in their respective centres. Indwelling urethral catheters are periodically replaced about 30 days of use; to not interfere with current clinical practice, the policy of each centre (or the investigator criteria) for catheter replacement and removal is considered valid for this study.

##### Blinding

This study is not blinded and the patient and health staff will know whether the patient is allocated to intervention or control arms. Differences in the shape and color of urinary catheters made study blinding too complex. To prevent bias the primary outcome will be evaluated by a data adjudication committee blinded to the intervention.

##### Randomization

The random sequence for the two comparison groups is computer generated ensuring equal allocation ratio. Blocked stratification for study centre is used to ensure that the two groups do not differ between centres. On-line central randomization ensures that intervention allocation is properly concealed.

Due to the characteristics of the included patients, it would be possible that they still require the use of an indwelling urinary catheter after their participation in the study. The study allows the participants to be randomized more than once if all the inclusion and exclusion criteria are still valid. Between first and subsequent participation in the study, patients should use standard catheters.

##### Data collection

All data is collected through an on-line electronic data capture system (http://ensayoescale.com). Secure access to this on-line platform is restricted to each centre investigator and designed collaborators and ensured by individual passwords.

The study on-line platform collects from each participant the inclusion and exclusion criteria, baseline clinical and demographic data, the allocated intervention (size, reference number, and whether the aseptic conditions are observed), any sign and symptom which may be suggestive of acute UTI, results from urine and/or blood analysis (if performed), antimicrobial susceptibility test (if performed), any treatment given, adverse events as well as the data and reason for urinary catheter replacement or removal.

**Figure 1:**
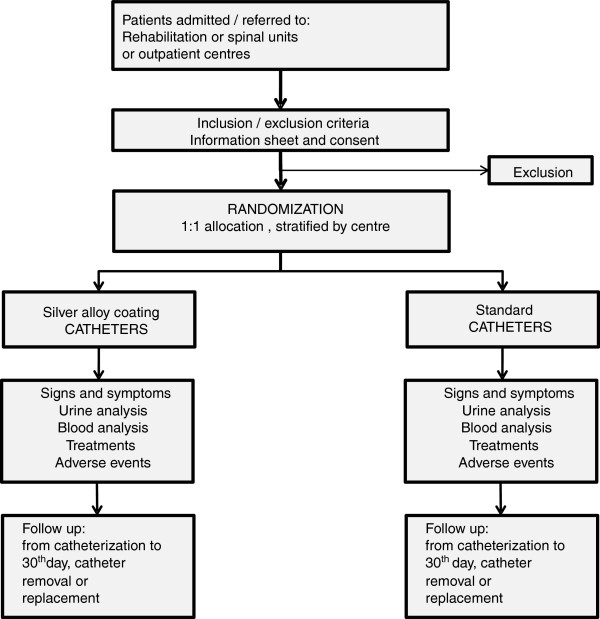
Study flow diagram.

See Figure 1 for the study flow diagram.

#### Measures

##### Socio-demographic and baseline clinical data

The following data are registered: date of birth, gender, level of spinal cord injury, traumatic or medical causes of spinal cord injury, ASIA scale [12], time with indwelling urethral catheter or first-ever urethral catheterization, presence of diabetes mellitus and medical treatments history (including treatments that may increase risk of infection).

##### Primary outcome

The primary outcome is the development of a UTI related to urinary catheterization.

The patient must have at least one sign or symptom suggestive of UTI (see Table 2) with no other recognized cause and a positive urine culture with no more than 2 species of microorganisms. The study does not establish a specific threshold of uropathogen colony count criteria for the diagnosis of UTI and use each centre criteria.

**Table 2:** Signs and symptoms suggestive of urinary tract infection

Increased spasticity	Costovertebral pain or tenderness
Fever (body temperature > 38°C)	Foul-smelling urine
Chills	Change in urine colour
Profuse sweating	Change in voiding patterns
Dysuria	Autonomic dysreflexia
Suprapubic pain or tenderness	Any other sign or symptom that to the investigator criteria may be suggestive of UTI
Abdominal pain or tenderness	

An UTI is considered associated with the use of study catheter if the specimen collection is performed at any time from catheterization procedure and 30^th^ day, catheter removal or catheter replacement (whichever occurs first).

It was considered that knowing if one type of urinary catheter or another may reduce symptomatic UTI is critical for clinical decisions. Many other studies considered asymptomatic bacteriuria (ASB) as primary outcome but ASB is very common in SCI patients, especially in those using indwelling catheters, and it appears to have no long-term squeals [13].

##### Secondary outcomes

Asymptomatic UTI, bacteremic UTI, and adverse events related to catheterization procedure are considered secondary outcomes.

Patients with an asymptomatic UTI are those with no signs or symptoms but with a positive urine culture with no more than 2 species of microorganisms in an specimen collected at any time from catheterization procedure and 30^th^ day, catheter removal or catheter replacement (whichever occurs first).

Patients with bacteremic UTI are those fulfilling the primary outcome and with a positive blood culture with at least 1 matching uropathogen microorganism to the urine culture.

The study measures the direct costs attributable to the use of intervention or control urinary catheters and their complications.

Any untoward medical occurrence in a patient using catheters with antiseptic silver alloy coating or standard catheters under study are also recollected.

### Statistical analysis

Primary efficacy analysis will be performed on the intent to treat population set, including those participants correctly randomized and willing to participate in the study. Per protocol analysis will also be performed including those participants correctly randomized, willing to participate and with no major protocol violations. A further exploration of the sensitivity will be performed with these two sets of population to check they lead to the same conclusions. Primary and secondary outcome analyses including dichotomous data will be analyzed using Chi-squared test (or Fisher’s exact test if analysis sample size is small). Socio-demographic and baseline clinical data will be described providing mean and standard deviation (or median and range) and compared using ANOVA test (or Kruskall-Wallis test) depending whether the data fulfil the application requirements.

In addition a regression analysis depending on gender, lesion level, re-inclusion and indwelling urethral catheterization carriers, first-ever catheter or another significant or clinical relevant outcome will be considered. No interim analysis is planned.

The level of significance will be the usual 5% (alpha = 0.05), two-sided. All the analysis will be performed with SPSS (V19.0).

Power calculation: The sample size was calculated on the assumption that 24% of patients will suffer from an UTI related to urinary catheterization, and a relative reduction of 35% with the use of catheters with antiseptic silver alloy coating, which is considered clinically relevant. A total sample size of 742, 371 in each group (allowing for 5% attrition) have a minimum of 80% power to detect these differences between intervention groups.

There is very limited information on the overall incidence of UTI in SCI patients using indwelling catheters, most coming from observational studies that included patients using different voiding methods, with a variety of risk factors, using heterogeneous definitions of UTI. In addition follow-up periods and incidence measures used varied broadly. We thus considered, conservatively, that almost one quarter of participants will suffer from an UTI [14,15].

## Discussion

This study is a randomized controlled trial to assess the incidence of symptomatic UTI in patients with SCI and needing an indwelling urinary catheter. The trial is designed to provide an adequate answer whether patients requiring long periods of catheterization might benefit from antiseptic-impregnated catheters (silver and other noble metals coating). Patients will be assessed during 30 days or until catheter removal. Signs and symptoms suggestive of UTI and urine cultures will be collected for the definition of a clinically relevant primary outcome. Other outcomes such as bacteremic UTI, adverse events and costs will be also accounted.

Coating urinary catheters with silver alloy and other noble metals as well as with antibiotics seem to be a promising intervention to reduce hospital-acquired infections, as described in a systematic review of randomized clinical trials [4]. This review included studies using short-term catheterization and pooled the results of a surrogate outcome such as bacteriuria. One recent clinical trial did not show clinically relevant differences between antiseptic-impregnated catheters (silver alloy or antimicrobial) to standard ones in 7102 adults with acute pathologies requiring a short-term urinary catheterization [6]. This large and pragmatic clinical trial defined catheter-associated UTI as the presence of participant-reported suggestive symptoms and clinician prescription of antibiotic for a UTI at any time up to 6 weeks after randomization. Even though this unusual definition, in patients carrying a catheter for an average of two days the results did not support the routine use of antiseptic-impregnated catheters. However, these evidences are not directly applicable to SCI patients.

Our study has several methodological strengths such as an accurate method of randomization, concealment allocation and a data adjudication committee blinded to the intervention. It also needs to be emphasized that the primary objective of our study has not been previously evaluated in SCI population and is based in a sound primary outcome which is critical for decision-making. In addition, the study is designed to find a clinically meaningful reduction in the relative incidence of UTI of 35%. This figure is translated to an absolute reduction of 84 fewer UTI per 1000 patients using indwelling catheters. Conversely, if the study does not rule out irrelevant or no benefit from the use of silver-alloy coating urinary catheters, most SCI patients requiring indwelling urethral catheterization would use the standard catheters which cost is 10 times less as compared to silver-alloy ones.

There are also several potential limitations of the trial. The most important one is its blinded nature. Patients and health staff will know the allocated intervention, thus they might become more prone to report sign, symptoms as well as to ask for urine or blood tests if they know that a standard catheter is used. This source of bias would be mitigated with the data adjudication committee blinded to the intervention. Another limitation is the UTI definition. Even though the Centers for Disease Control and Prevention [16] recognizes that subjects with at least one of the following signs or symptoms (fever (>38°C), suprapubic tenderness, or costovertebral angle pain or tenderness) and a positive culture suffer from a symptomatic UTI, the sensitivity and specificity of urinary symptoms in SCI patients is very poor [13,17]. The diagnosis of UTI in SCI patients is based on the combination of symptoms and signs, which are often non-specific. The signs and symptoms criteria for UTI in this study are adapted from the National Institute on Disability and Rehabilitation Consensus Statement [18], which together with other clinical and laboratory data will be the basis for data adjudication committee to decide if a patient suffered from an UTI.

### Conclusion

We present the protocol of an open label, randomized controlled clinical trial to determine the effectiveness of urinary catheters with antiseptic silver alloy coating on the reduction of symptomatic UTI in SCI patients with indwelling urethral catheter. The urinary catheters with antiseptic silver alloy coating are compared to standard urinary catheters (with no antiseptic coating) during a period up to 30 days. This study will provide clear information about the clinical effectiveness of silver-alloy catheters to guide its use in a population requiring long-term catheterization.

## Abbreviations

ASIA: American spinal injury association; UTI: Urinary tract infection; ANOVA: Analysis of variance; SCI: Spinal cord injury; ASB: Asymptomatic bacteriuria.

## Competing interests

Manuel Bea-Muñoz received funding from *Coloplast* and *Hollister* for his participation in conferences and courses. He received fees from *Wellspect Halthcare* for conferences talks. The rest of authors declare that they have no competing interests.

## Authors’ contributions

XB, DR, MJM conceived of the study, participated in its design and coordination and drafted the manuscript. MLJ, JMB, SSB, CMA, MB, SM, AB, JRE, LL, ME and IA made substantial contributions to design, revised the manuscript critically for intellectual content and have given final approval of the version to be published. All authors read and approved the final manuscript.

## Pre-publication history

The pre-publication history for this paper can be accessed here:

http://www.biomedcentral.com/1471-2490/13/38/prepub

## Supplementary Material

Additional file 1: Table S1 ESCALE Group. Click here for file
