# Supplementary material for: A randomized controlled trial to assess the efficacy and cost-effectiveness of urinary catheters with silver alloy coating in spinal cord injured patients: trial protocol
Source: BMC Urol. 2013 Jul 30;13:38. doi: 10.1186/1471-2490-13-38 (PMC3735409; doi:10.1186/1471-2490-13-38)
Supplement: Additional file 1: Table S1 — ESCALE Group. [file 1471-2490-13-38-S1.docx]

Additional file 1

ESCALE Group

| Principal Investigator | Xavier Bonfill Cosp | Iberoamerican Cochrane Centre |
| --- | --- | --- |
| Study Coordination | David Rigau | Iberoamerican Cochrane Centre |
|  | María José Martínez Zapata | Iberoamerican Cochrane Centre |
| Investigators  *(alphabetical order)* | Carolina María Alemán Sánchez | Complejo Hospitalario Universitario Insular – Materno Infantil de Canarias |
|  | Cruz Almuiña Díaz | Complexo Hospitalario Universitario A Coruña |
|  | Ervin Amaya | Hospital de Neurorrehabilitación - Instituto Guttmann |
|  | Lourdes Antolín Horno | Hospital Universitario Miguel Servet |
|  | Enrique Bárbara Bataller | Complejo Hospitalario Universitario Insular – Materno Infantil de Canarias |
|  | Juana María Barrera Chacón | Hospital Universitario Virgen del Rocío |
|  | Manuel Bea Muñoz | Hospital Universitario Central de Asturias |
|  | Jesús Benito | Hospital de Neurorrehabilitación - Instituto Guttmann |
|  | Aníbal Blanco Vivo | Hospital Universitario Puerta del Mar |
|  | Albert Borau Duran | Hospital de Neurorrehabilitación - Instituto Guttmann |
|  | Nora Cívicos Sánchez | Hospital Universitario Cruces |
|  | Montserrat Cuadrado Rebollares | Hospital Universitario Cruces |
|  | Manuel Florencio de la Marta García | Hospital Nacional de Parapléjicos de Toledo |
|  | Juan Gabriel de los Ríos | Hospital de Neurorrehabilitación - Instituto Guttmann |
|  | Juan Ramón Espinosa Quiros | Hospital Universitario Puerta del Mar |
|  | Manuel Esteban Fuertes | Hospital Nacional de Parapléjicos de Toledo |
|  | Mª Elena Ferreiro Velasco | Complexo Hospitalario Universitario A Coruña |
|  | Inmaculada García Obrero | Hospital Universitario Virgen del Rocío |
|  | Mónica Garrán Díaz | Hospital Universitario Central de Asturias |
|  | Mónica González Nuño | Hospital Universitario La Paz |
|  | Ena Eva Granados Matute | Hospital Universitario Virgen del Rocío |
|  | Mari Paz Herederos | Hospital de Neurorrehabilitación - Instituto Guttmann |
|  | Ricardo Hermida Pérez | Hospital Universitario Puerta del Mar |
|  | María Luisa Jauregui Abrisqueta | Hospital Universitario Cruces |
|  | Luis Ledesma Romano | Hospital Universitario Miguel Servet |
|  | Inés Lodeiro Mendieta | Hospital Universitario Cruces |
|  | Antonio López García Moreno | Hospital Nacional de Parapléjicos de Toledo |
|  | Bosco Méndez Ferrer | Hospital Universitario Virgen del Rocío |
|  | Antonio Montoto Marqués | Complexo Hospitalario Universitario A Coruña |
|  | Susana Moraleda Pérez | Hospital Universitario La Paz |
|  | Montse Morcillo | Hospital de Neurorrehabilitación - Instituto Guttmann |
|  | Teresa Ayala Ortiz de Solorzano | Hospital Universitario Miguel Servet |
|  | Ana Luz Peña Florez | Hospital Universitario Miguel Servet |
|  | Francisco Pineda Rivero | Hospital Universitario Miguel Servet |
|  | Ricardo Piñeiro Rojas | Hospital Universitario Virgen del Rocío |
|  | Fátima Quintana Suárez | Complejo Hospitalario Universitario Insular – Materno Infantil de Canarias |
|  | Francisco Reche Pérez | Hospital Universitario Puerta del Mar |
|  | Antonio Rodríguez Sotillo | Complexo Hospitalario Universitario A Coruña |
|  | Francisco Javier Salguero Ruiz | Hospital Universitario Puerta del Mar |
|  | Sebastián Salvador de la Barrera | Complexo Hospitalario Universitario A Coruña |
|  | Catalina Silvestre Agudelo | Hospital Universitario Miguel Servet |
|  | Joan Vidal | Hospital de Neurorrehabilitación - Instituto Guttmann |
|  | Miguel Virseda Chamorro | Hospital Nacional de Parapléjicos de Toledo |
|  | María José Zarco Periñan | Hospital Universitario Virgen del Rocío |
